# Postoperative results of laparoscopic lateral suspension operation: A clinical trials study

**DOI:** 10.3389/fsurg.2023.1069110

**Published:** 2023-01-26

**Authors:** Şerif Aksin, Cengiz Andan

**Affiliations:** ^1^ Obstetrics and Gynecology Department, Fakulty of Medicine, Siirt University, Siirt, Turkey; ^2^ Obstetrics and Gynecology Department, Şişli Hamidiye Etfal Training and Research Hospital, İstanbul, Turkey

**Keywords:** laparoscopy, lateral suspension, prolapse uteri, operation, results

## Abstract

**Background:**

Pelvic organ prolapse surgery carries potential risks, and Laparoscopic lateral suspension (LLS) surgery is being performed in increasing numbers with advances in minimally invasive surgery. Our study aims to report the postoperative results of LLS operations.

**Patients and Methods:**

41 patients at POP Q stage 2 and above underwent LLS operations in a tertiary center between 2017 and 2019. Postoperative patients 12 (12–37) months and older were evaluated in terms of anterior and apical compartments.

**Results:**

In our study, laparoscopic lateral suspension (LLS) was applied to 41 patients. The mean age of all patients was 51.45 ± 11.51, and the operation time was 71.13 ± 18.70 min, The mean hospital stay was 1.35 ± 0.4 days. The apical compartment success rate was 78% and the anterior compartment success rate was 73%. In terms of patient satisfaction, 32 (78.1%) patients were satisfied, While 37 (90.1%) patients did not have abdominal mesh pain, 4 (9.9%) patients had mesh pain. Dyspareunia was not observed.

**Conclusions:**

Laparoscopic lateral suspension in pop surgery; Considering the success rate below expectation, some patient groups can be applied as an alternative surgical method.

## Introduction

Pelvic organ prolapse (POP) is the downward descent of the anterior and posterior wall of the vagina, uterus, and apex of the vagina (1). Women in the United States have a 13% lifetime risk of having POP surgery (2). The prevalence of pelvic organ prolapse surgery ranges from 6% to 18%. The incidence of POP surgery ranges from 1.5 to 1.8 per 1,000 women and peaks among women aged 60 to 69 years (3).

Although the weakening of the pelvic ligaments plays an important role in the pathophysiology of POP, neither its etiology nor its pathophysiology is fully understood (4). Parity, vaginal delivery, age, and BMI (body mass index) are risk factors for POP, and the preoperative stage is a risk factor for POP recurrence (5).

In the treatment of POP, mild and moderate patients can be treated using conservative methods, such as lifestyle changes, pelvic floor exercises, and use of vaginal pessary (6). Surgical treatment is recommended for severely symptomatic patients and when primary intervention has failed (7).

Abdominal and vaginal approaches are used in surgical treatment. Laparotomy/laparoscopic/robotic sacrohysteropexy, uterosacral ligament suspension, and sacrospinous ligament fixation are operations that are commonly performed. The use of vaginal mesh is controversial, but it may have a place in the repair of recurrent prolapses, especially in the anterior compartment (8). The uterus has been found to function only as a passive structure rather than a cause in the development of POP. Therefore, uterus-sparing surgery and sacrohysteropexy have aroused immense interest among surgeons. Unfortunately, concomitant hysterectomy remains a highly common procedure (9).

Laparoscopic and robotic approaches have been preferred over the abdominal approach, with advances in minimally invasive surgery. Laparoscopy yields comparable anatomical and functional results with less blood loss, faster recovery, and lower overall complication rates. Although laparoscopic sacrohysteropexy is the operation of choice in POP surgery, there is a risk of intraoperative urological, gastrointestinal, hemorrhagic, and neural complications (10).

Therefore, surgeons have shifted to alternative operations that are effective but easier and with less potential for intraoperative complications. Laparoscopic lateral suspension has gained acceptance among surgeons in increasing numbers in the last decade. In this study, we aimed to evaluate our results regarding laparoscopic lateral suspension operations.

## Materials and methods

Diyarbakır Gazi Yaşargil Training and Research Hospital Clinical Research Ethics Committee approval no: (2021/905). Clinical trials no: NCT04178083.

Our study included 41 patients who underwent laparoscopic lateral suspension operation with the diagnosis of uterine prolapse in our hospital between 01.05.2017 and 31.12.2019. Cases with apical uterine prolapse at Stage 2 and above were included in the study according to the POP Q staging system. Anatomical points and landmarks for POP–Q system examination: Aa (point A anterior**)**, Ap (point A posterior), Ba, (point B anterior); Bp(point B posterior); C (cervix or vaginal cuff); D (posterior fornix) (if cervix is present; TVL (total vaginal length). Aa, Ba, C, Ap, Bp, as well as TVL values in POP Q scoring were noted before the operation. Patients over 70 years of age, severe cardiovascular patients, and pregnant women were excluded from the study. All operations were performed by two surgeons with optimal skills in laparoscopic surgery.

After the operation, patients (12 to 36 months were evaluated by a gynecologist who did not participate in the operations) at least 12 months after the operation. Age, parity, operation history, previous prolapse surgery, operation time, hospital stay, estimated blood loss, prolapse degree, anterior and posterior compartments and surgeries for them, accompanying operations, Visual Analog Scale (VAS), and general health satisfaction questionnaire results were evaluated. Anatomical points Aa, Ba, C, Ap, Bp, TVL values were measured in POP Q scoring for apical, anterior, and posterior compartment defects. Patients below Stage 2 according to POP Q scaling were regarded as anatomically successful.

### Operation techniques

After a 10 mm infraumbilical port was placed, two 5 mm lateral ports were placed 4 cm superior to both Spina iliaca anterior superior. An ipsilateral 5 mm port was placed on the right side. After preparing a V-shaped polypropylene mesh with a length of 25 cm with arms and a size of 5 * 5 cm with a base, it was inserted into the abdomen using a 10 mm trocar.

**Figure 1:**
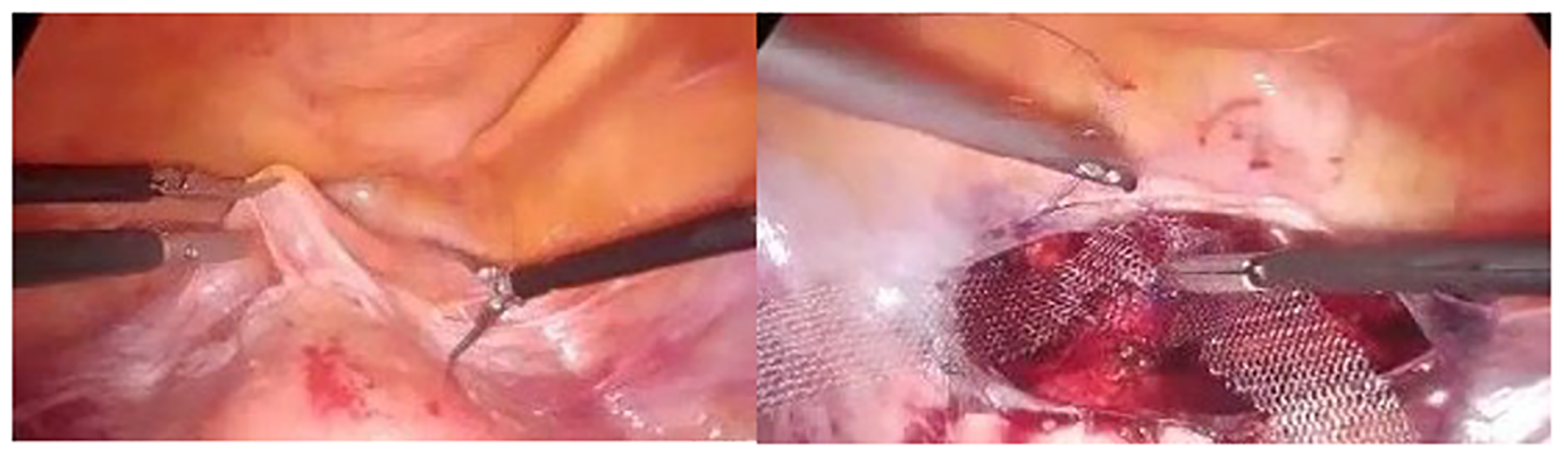
Opening the vesicouterine peritoneum and suturing the V-shaped mesh to the cervico-isthmic area.

The anterior cervical area and isthmus uteri was exposed by dissecting the vesicouterine peritoneum. A mesh base of 4*4 cm dimensions was fixed to the isthmus uteri region with intracorporeal sutures with 2–0 prolene (Figure 1). We make adequate fixation by applying the front strip of the mesh well.

**Figure 2:**
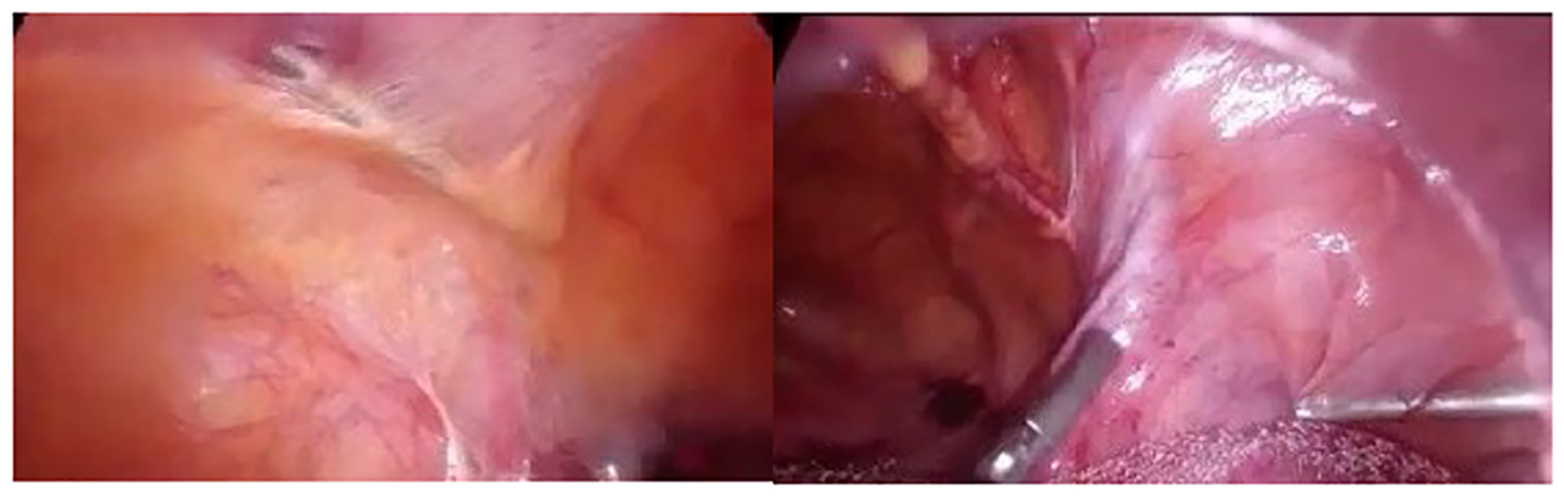
Reaching the mesh arms with the subperitoneal from the modified port site of the laparoscopic portegu.

Subsequently, the trocars were removed from the modified port locations, the lateral portegu was advanced under the sub-peritoneum, and the cervical area was reached under the round ligament (Figure 2).

**Figure 3:**
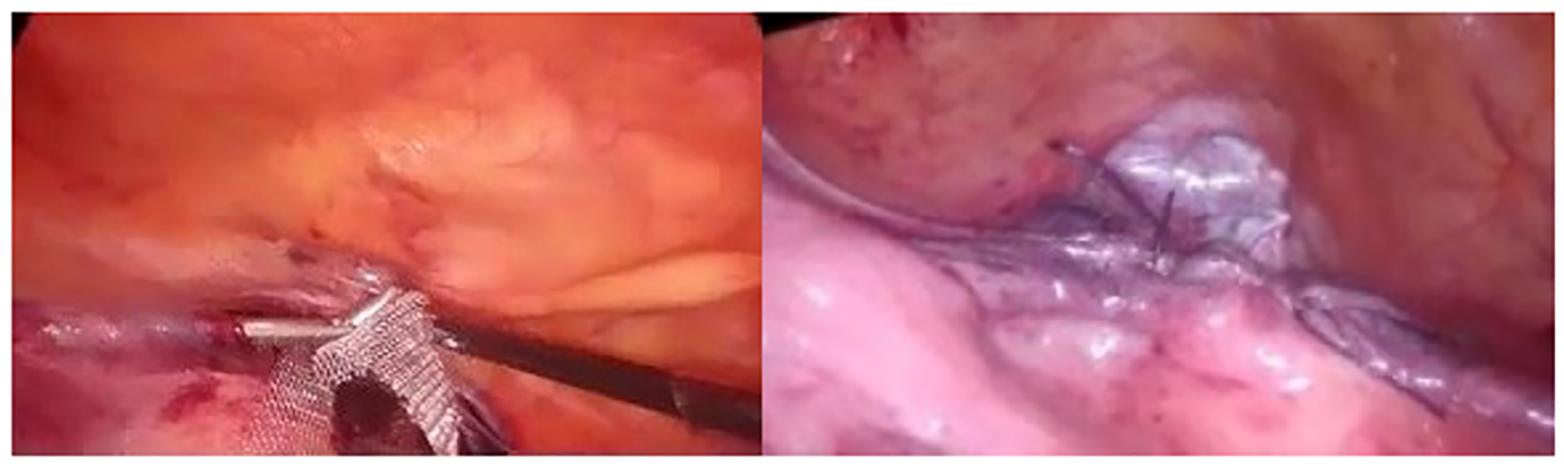
Vesicouterin closure of the peritoneum.

The removed ports were repositioned by sliding the lateral ports over the mesh. The vesicouterine peritoneum was closed with 2–0 vicryl (Figure 3). The bilateral mesh ends were cut at the skin level. We leave both lateral suspensions tension free. After the gas in the abdomen is evacuated, the mesh ends are released.

### Statistical analysis

In the descriptive statistics of continuous variables, mean, standard deviation, median, minimum, and maximum values are given, and in the definition of categorical variables, frequency (n) and percentage (%) values are provided. IBM SPSS.23 program was used in all analyses.

## Results

In our study, laparoscopic lateral suspension (LLS) was applied to 41 patients. The average age was 51. 13 patients underwent additional incontinence surgery. The mean hospital stay was 1.35 ± 0.4 days. At the end of one-year follow-up, mesh pain in the abdominal wall was evaluated. While 37 (90.1%) patients did not have mesh pain, 4 (9.9%) patients had mesh pain. At the end of the first year, the uterus level (pop-Q C) did not improve in 9 (24.3%) patients, while the uterus level was at the desired level in 32 (78%) patients. The anterior component (pop-Q Ba) persisted in 11 (27%) patients and resolved in 30 (73%) patients. The rectocele component (pop-Q Bp) persisted in 17 (41.4%) patients and improved in 24 (56.6%) patients. In terms of patient satisfaction, 32 (78.1%) patients were satisfied, while 9 (21.9%) patients were not satisfied. Finally, the uterus was in the retroverted position in 36.5% of the patients under the postoperative USG controls (Table 1).

**Table 1:** Medical and sociodemographic characteristics of patients measured as categorical variables.

Parameter		Frequency	Percent
**The surgery performed**			
LLS		41	
LLS + incontinence surgery		13	
**POP Q**	**preoperative**		
Stage 0		0	
Stage 1		0	
Stage 2		17	41.5
Stage 3		17	41.5
Stage 4		7	17
**Hospital stay time**			
1		30	73.3
2		11	26.7
**Abdominal mesh pain**			
Yes		4	9.9
No		37	90.1
**Anterior compartment**			
Fail		11	27
Successful		30	73
**Apical compartment**			
Fail		9	22
Successful		32	78
**Patient satisfaction**			
Satisfied		32	78.1
Unsatisfied		9	21.9
**Uterus position**			
Antevert		26	63.5
Retrovert		15	36.5
**Dyspareunia**		0	0

Table 2 indicates the mean, standard deviation, median, minimum, and maximum values of parameters measured as continuous variables of the patients involved in the study. The mean age of all patients was 51.45 ± 11.51, and the operation time was 71.13 ± 18.70 min, as revealed in Table 2. The Pop Q anterior compartman scores (Ba) before and after the operation were found to be 2.90 ± 1.42 and −1.48 ± 1.93, respectively. Pop Q posterior compartman scores (Bp) before and after the operation were found to be 1.03 ± 2.79 and −0.96 ± 2.84, respectively. Finally, the Pop Q cervix value before the operation was 3.81 ± 2.91, and the Pop Q cervix value after the operation was −5.52 ± 3.90.

**Table 2:** The mean, standard deviation, median, minimum, and maximum values of the medical and sociodemographic characteristics of the patients measured as continuous variables.

Parameter	*n*	Med. ± SD.	Median (Min. − Max.)
Operation time	41	71.13 ± 18.70	70.00 (45.00–120.00)
Pop Q anterior compartman (preop)—Ba	41	2.90 ± 1.42	3.00 (1.00–6.00)
Pop Q anterior compartman (postop)—Ba	41	−1.48 ± 1.93	−2.00 (−3.00–4.00)
Pop Q posterior compartman (preop)—Bp	41	1.03 ± 2.79	0.00 (−2.00–6.00)
Pop Q posterior compartman (postop)—Bp	41	−0.96 ± 2.84	−2.00 (−3.00–6.00)
Pop Q cerviks (preop)—C	41	3.81 ± 2.91	3.00 (−4.00–8.00)
Pop Q cerviks (postop)—C	41	−5.52 ± 3.90	−6.00 (−7.00–6.00)

## Discussion

The history of laparoscopic sacrohysteropexy/colpopexy has been continuing for over 30 years (11). Laparoscopic lateral suspension was first described by Cornier et al. and then developed by Dubuisson (12, 13). Laparoscopic sacrohysteropexy/colpopexy is regarded as the gold standard in uterine prolapse surgery (14). However, it keeps other surgical approaches alive due to the variety of reported complications. Vaginal mesh surgery has a morbidity rate (especially in sexual activity) that is too high to be considered the best surgical treatment. However, this problem will probably find another answer in the future, when all the work on the components of the meshes is finished (15, 16).

In our study, laparoscopic lateral suspension anatomical efficiency was found in the apical compartment, 77.7% (Pop Q (preop)-C, 3.81 ± 2.91, Pop Q (postop)—C, −5.52 ± 3.90). In their highest volume study (n:417), Veit-Rubin et al. reported anatomical success rates at the end of 12 months as 91.6% for the anterior compartment and 93.6% for the apical compartment. They reported that a randomized controlled trial must be an alternative to sacrohysteropexy in the treatment of POP (17). Dubuisson JB, one of the architects of this surgical technique, observed prolapse recurrence in 13.76% of patients in 218 disease series (18). In a study of 88 patients with a mean follow-up of 3.4 years, Chatziioannidou K et al. Reported that the objective cure rate as 87.3%, and the reoperation rate for recurrence was 5.1% (19). In a prospective study of 120 patients, Mereu et al. reported that the anatomical success rate was 94.2% for the anterior compartment, 94.9% for the apical compartment, and POP-Q recurrences were concentrated in the first six months (20). In a prospective study in which Yassa et al. followed 17 patients for 24 months, the anatomical cure rate was 100% for the apical compartment and 88.2% for the anterior compartment. They reported that Ba and C scores improved significantly, and nocturia symptoms improved (*p* = 0,053) (21). Martinello et al. reported success rates of 100% for the apical compartment, 92% for the anterior compartment, and over 80% for the patient's self-perception of their own health, in a retrospective study of 48 patients followed at 12 months (22). Campagna et al. reported an overall anatomical success rate of over 90% in the apical compartment and more than 88% in the anterior compartment in a systematic review of 1,066 operations on LLS (23).

Considering the anterior compartment success rates in our study, the anterior compartmen continued in 29.3% of patients and resolved in 70.7%. (Pop Q—preoperative Ba score 2.90 ± 1.42 and Pop Q postoperative—Ba score −1.48 ± 1.93). Studies have reported highly satisfactory results regarding the anterior compartment in laparoscopic lateral suspension (23, 24). It was observed that the need for a second intraoperative operation for the anterior compartment defect declined in the vaginal examination immediately after the LLS operation. Veit et al. reported that particularly the uterine-sparing approach had superior anatomical results for the anterior compartment in LLS (25).

Mulayim et al. developed a new technique in 2019 by modifying the mesh type and port location. In our study, we placed the lateral ports at the mesh locations specified in the classical method. After the mesh base is fixed to the cervix, we pull the mesh arms with the help of a laparoscopic portegu after the mesh base is fixed on the lateral port sites, which we placed 4 cm superior to the bilateral crista iliaca anterior superior. No vascular and neural injuries related to the modified port sites were observed. Surgeons should be careful when placing lateral ports due to the reduced bowel distance, but the risk is reduced since this procedure is performed under umbilical camera surveillance (26).

In our study, the operation time was 71.13 ± 18.70 min, and the mean hospital stay was 1.35 ± 0.4 days. No major complications were observed in the operations. Laparoscopic sacrohysteropecxy operation has a lengthy learning process, long operation time, and severe complications. In this regard, laparoscopic lateral suspension appears to be more advantageous. Our operation method, which combines two incisions, contributes to this reduction. Less operation time and less complication rates relieve surgeons (24).

However, mesh-related problems are a matter of curiosity due to the long arms of the mesh used. Mesh erosion was not reported in our study. Mesh pain with a VAS score of 3 to 5 was reported in four (12.9%) patients. Dällenbach Pet et al. conducted a study of 133 cohorts in which they specifically investigated mesh erosion in laparoscopic lateral suspension and had a mean follow-up of 82.3 months. They reported that the risk of mesh erosion is low (3.8%) and can be further reduced by using appropriate mesh material and identifying certain patient characteristics, such as reducing smoking (27).

In our study, a patient with a pelvic kidney was treated with laparoscopic lateral suspension. Additionally, a 67-year-old comorbid patient with a previous history of laparoscopic sacrocervicopenia, whose promontory was difficult to dissect due to fibrosis, was successfully treated with laparoscopic lateral suspension. Surgeons performing prolapse surgery should be prepared for intraoperative unpredictable problems (obesity, adhesions, sigmoid megacolon, vascular variations). In laparoscopic POP surgery, it is useful for surgeons to know about LLS in difficult intraoperative situations and as a preoperative secondary surgery. Okada Y et al. stated that it would be beneficial for surgeons who perform laparoscopic cervicopexy to know the laparoscopic lateral suspension procedure in terms of intraoperative transformation (28).

No dyspareunia was reported in our study. In addition, postoperative uterine localization was retroverted in 35% of the patients. There are insufficient studies on dyspareunia and sexual life due to the use of mesh and the redesign of the uterine anatomical location due to suspension. Milani et al. reported a 46-year-old patient who presented with severe pelvic pain and inability to have sexual intercourse after LLS operation and was treated with mesh excision. Pulatoğlu et al. reported that the vaginal ax axis was close to normal, and the uterovaginal angles did not change in patients who underwent LLS (29, 30).

In terms of the limitations of our study, our study is not a randomized controlled study. The sample size is comparatively smaller. The strength of our study is that it contributes to the limited literature on the subject. The operations were performed by two surgeons with optimal skills. Although the operations were retrospective, the prolapse evaluations were evaluated prospectively and blinded by an expert who did not participate in the operations.

## Conclusion

Prolapse surgery offers its own difficulties. The management of relapses and the search for alternatives to intraoperative challenges keep it alive. LLS is performed by increasing number of surgeons due to developments in minimal invasive surgery. LLS operations, intraoperative difficulties occur and anatomical variations and other standard methods are not used. Laparoscopic lateral suspension has a success rate below expectation in pop surgery. It can be considered as an alternative method in appropriate patient groups.

## Data Availability

The original contributions presented in the study are included in the article/Supplementary Materials, further inquiries can be directed to the corresponding author/s.
